# Incidence and Clinical Presentation of Pre- and Postoperative Seizures in Patients With Posterior Fossa Meningiomas

**DOI:** 10.7759/cureus.52474

**Published:** 2024-01-18

**Authors:** Lukas Goertz, Nora Bernards, Hannah Muders, Christina Hamisch, Roland Goldbrunner, Boris Krischek

**Affiliations:** 1 Department of Radiology, University of Cologne, Faculty of Medicine and University Hospital Cologne, Cologne, DEU; 2 Department of General Neurosurgery, University of Cologne, Faculty of Medicine and University Hospital Cologne, Cologne, DEU

**Keywords:** surgery, seizures, posterior fossa, meningioma, craniotomy

## Abstract

Introduction: Seizures are a common symptom of supratentorial meningiomas with pre- and postoperative seizure rates of approximately 30% and 12%, respectively, especially in parasagittal and convexity meningiomas. Less is known about the association between seizures and posterior fossa meningiomas. This study evaluates the prevalence, potential causes, and outcomes of seizures in patients who have undergone surgery for posterior fossa meningioma.

Methods: This is a retrospective, observational, single-center study of consecutive patients who underwent surgical resection of posterior fossa meningiomas between 2009 and 2017. We retrospectively identified patients with seizures and analyzed patient demographics, tumor characteristics, and procedural characteristics.

Results: A total of 44 patients (mean age: 59.8 ± 13.5 years) were included. Twenty-six tumors were located at the cerebellar convexity and tentorium (59.1%), 12 at the cerebellopontine angle (27.3%), four at the clivus (9.1%), and two at the foramen magnum (4.5%). Seizures were the presenting symptom of cerebellar meningioma in two patients. Patients were seizure-free after surgery. Three patients had their first seizure after surgery (interval between surgery and first seizure: two days to 17 months). Analysis of these three patients revealed possible causes of postoperative seizures: radiation necrosis and edema, hyponatremia, and preoperative hydrocephalus. In all patients with postoperative seizures, long-term seizure control was achieved with the administration of antiepileptic drugs.

Conclusions: The incidence of seizures in patients with posterior fossa meningiomas is relatively low. Antiepileptic drugs can help to achieve seizure control.

## Introduction

Intracranial meningiomas account for approximately 35% of all primary brain tumors [1]. Seizures are a common symptom associated with intracranial meningiomas. In some patients, seizures are the presenting symptom of meningioma, while other patients develop seizures after surgical resection of the tumor [2]. In general, seizures are treated with antiepileptic drugs (AEDs). However, long-term seizure control is not always achieved, and AEDs may have significant side effects, such as lack of tolerability and impairment of neurocognitive function [3-5]. In recent years, several studies have analyzed the risk factors associated with pre- or postoperative seizures in meningioma patients [2,6-9,10]. In general, it is expected that seizures are not frequent in patients with posterior fossa meningiomas due to the distance to the epileptogenic cortex [11]. Therefore, most studies have focused on supratentorial meningiomas [7,10,12-14], and results on the association between posterior fossa meningiomas and seizures have not yet been published. The purpose of the present study was to retrospectively evaluate the prevalence of pre- and postoperative seizures in patients undergoing surgery for posterior fossa meningioma and to identify potential causes and clinical outcomes.

## Materials and methods

This is a retrospective, single-center, observational study conducted at the University Hospital of Cologne, Germany. The retrospective, anonymized data collection was approved by the local ethics committee (number: 20-1409), and informed consent was obtained from all patients before surgery. Due to the retrospective, observational design of this study, separate institutional approval was not required.

Inclusion criteria

All consecutive patients who underwent surgery at the authors' institution for intracranial meningioma between January 2009 and December 2017 were identified by medical chart review. All patients older than 18 years who underwent surgery for newly diagnosed posterior fossa meningioma were identified and included in this study. There were no further exclusion criteria. Based on the classification of Castellano and Ruggiero, we included cerebellar convexity and tentorial meningiomas, petroclival and clivus meningiomas, cerebellopontine angle meningiomas, and foramen magnum meningiomas [15]. For patients with repeat surgeries, only the first surgery was included in this study.

Procedural details

All procedures were performed under general anesthesia using an operating microscope. A suboccipital craniotomy was performed according to the tumor location (lateral and medial). After surgery, patients were admitted to an intensive care unit overnight. A plain cranial computed tomography (CT) scan was performed on the first postoperative day to exclude intracranial hemorrhage and major ischemia. Radiologic follow-up was performed periodically for three months after surgery by magnetic resonance imaging (MRI).

Data collection

The following data were retrospectively collected from the medical records: patient age, sex, preoperative Karnofsky performance score (KPS), initial symptoms at presentation, postoperative complications, the occurrence of pre- and postoperative seizures, seizure type (focal or generalized), the interval between surgery and postoperative seizures, the use of pre- and postoperative AEDs, tumor location, World Health Organization (WHO) tumor grade, the extent of tumor resection (Simpson grade). Tumor size and presence of peritumoral edema were assessed by preoperative MRI. For tumor size, the largest tumor diameter is reported. Peritumoral edema was graded on an ordinal scale (present or absent). Surgical details, including operative time and surgical approach, were obtained from the operative and anesthesia reports.

Statistical analysis

Baseline patient and meningioma characteristics were analyzed using descriptive statistics. Categorical variables were presented as numbers and percentages. Continuous variables were expressed as mean ± standard deviation. All calculations were performed with SPSS software (SPSS Statistics for Windows, version 25.0, IBM Corp., Armonk, NY). A p-value < 0.05 was considered statistically significant.

## Results

Patient and tumor characteristics

During the study period, a total of 365 patients underwent surgery for intracranial meningioma at our institution. We identified 44 patients with posterior fossa meningiomas (12.1%) and included these patients in our analysis. The mean age of the patients was 59.8 ± 13.5 years (range: 23-83 years), and 36 patients were female (81.8%). The median preoperative KPS was 90. Twenty-six tumors were located at the cerebellar convexity or tentorium (59.1%), 12 at the cerebellopontine angle (27.3%), four at the clivus (9.1%), and two at the foramen magnum (4.5%). Peritumoral edema was seen on preoperative MRI in 14 cases (31.8%). Histopathologic evaluation of tumor specimens revealed WHO grade 1 in 40 cases (90.9%), WHO grade 2 in three cases (6.8%), and WHO grade 3 in one case (2.3%). Baseline patient and aneurysm characteristics are shown in Table 1.

**Table 1:** Baseline patient and tumor characteristics SD: Standard deviation; KPS: Karnofsky performance score; WHO: World Health Organization.

Characteristics	Value
Age (years), mean ± SD	59.8 ± 13.5
≤60 years	25 (56.8%)
>60 years	19 (43.2%)
Gender	
Female	36 (81.8%)
Male	8 (18.2%)
Preoperative KPS, median	90
KPS 50	2 (4.5%)
KPS 70	3 (6.8%)
KPS 80	8 (18.2%)
KPS 90	13 (29.5%)
KPS 100	18 (41.0%)
Preoperative seizure	2 (4.5%)
Tumor location	
Cerebellar convexity/tentorium	26 (59.1%)
Cerebellopontine angle	12 (27.3%)
Foramen magnum	2 (4.5%)
Petroclival/clivus	4 (9.1%)
Peritumoral edema	
Yes	14 (31.8%)
No	28 (63.6%)
Data missing	2 (4.6%)
WHO grade	
WHO grade 1	40 (90.9%)
WHO grade 2	3 (6.8%)
WHO grade 3	1 (2.3%)

Operative details and complications

The mean operative time was 397 ± 259 minutes (range: 94-1126 min). Simpson grade 1 resection was achieved in two patients (4.5%), grade 2 in 26 patients (59.1%), grade 3 in seven patients (14.9%), and grade 4 in six patients (13.6%). Simpson grade data were missing in three patients (7.3%). Procedure-related complications occurred in seven patients (15.9%), including facial paralysis in two patients (4.5%), diplopia in two patients (4.5%), cerebrospinal fluid (CSF) fistula in two patients (4.5%), and ischemic infarction in one patient (2.3%). Postoperative seizures were observed in four patients (9.1%). Surgical details and complications are shown in Table 2.

**Table 2:** Surgical details and complications SD: Standard deviation; CSF: Cerebrospinal fluid.

Characteristics	Value
Operation time (min), mean ± SD	397 ± 259
*Extent of resection*	
Simpson 1	2 (4.5%)
Simpson 2	26 (59.1%)
Simpson 3	7 (15.9%)
Simpson 4	6 (13.6%)
Data missing	3 (7.3%)
*Complications*	
Facial paresis	2 (4.5%)
Diplopia	2 (4.5%)
CSF fistula	2 (4.5%)
Ischemic infarct	1 (2.3%)
Postoperative seizures	4 (9.1%)

Preoperative seizures

We identified two patients (4.5%) with preoperative seizures. In both cases, seizures were the presenting symptom of posterior fossa meningioma. Both patients were older than 60 years, female, and had a preoperative KPS of 100. The first patient had multiple focal seizures (Table 3), while the second patient had a single generalized seizure. The tumors were located at the cerebellar convexity and tentorium. No other intracranial meningiomas or other brain tumors were found. In the first patient, the meningioma was not considered to be the cause of the focal seizures. In the second patient, the tumor had a supratentorial part in contact with the left temporal lobe, which could have triggered the seizure. After tumor resection, both patients were seizure-free and did not require antiepileptic medication. Details of the two patients with preoperative seizures are shown in Table 3.

**Table 3:** Patient and tumor characteristics of two patients with preoperative seizures as presenting symptom for intracranial meningioma F: Female; KPS: Karnofsky performance score.

Case	Patient age/gender	Presenting symptoms	Number of preoperative seizures	Seizure type	Antiepileptic medication	KPS	Tumor location	Multiple brain tumors	Supratentorial tumor portion	Tumor size (cm)	Postoperative seizures
1	72 years/F	Seizures	Multiple	Focal (speech disorder, paraphasia)	No	100	Cerebellar convexity right	No	No	2.3	No
2	68 years/F	Seizure	1	Generalized	No	100	Tentorium left with supratentorial portion	No	Yes	2.1	No

Postoperative seizures

Three patients experienced postoperative seizures. All patients were female (age: 37-74 years). The meningioma was located at the cerebellar convexity in two cases and at the clivus in one case. The operation time ranged from 166 to 1000 minutes. Patient 3 had an infarction of the middle cerebellar peduncle, and the other patients had no procedural complications. One patient had a generalized seizure, and two had focal seizures. Two patients had seizures during hospitalization. The third patient had seizures at long-term follow-up. Several factors were identified in the patients as potential causes of postoperative seizures: Patient 1 had a WHO III meningioma and received postoperative tumor bed irradiation. The patient developed radiation necrosis associated with supra- and infratentorial edema. Patient 2 had a seizure due to postoperative hyponatremia (115 mmol/L). Patient 3 had a large clival tumor with consecutive brainstem compression and preoperative hydrocephalus. In all three patients, long-term seizure control was achieved with AED administration, using levetiracetam in two cases and lamotrigine in one case, both as monotherapy. There were no AED-related side effects. Details of all three patients with postoperative seizures are shown in Table 4.

**Table 4:** Patient demographics, tumor characteristics, and procedural specifics of four patients with postoperative seizures F: Female; N/A: Not available; g: Gram; mg: Milligram; d: Day.

Case	Patient age/gender	Presenting symptoms	Tumor location	Multiple brain tumors	Tumor size (cm)	Operation time (min)	Simpson grade	Procedural complications	Interval surgery - seizure	Seizure type	Antiepileptic medication	Potential cause for epilepsy
1	37 years/F	Dizziness	Cerebellar convexity left	No	3.2	325	2	No	19 months	Focal	Lamotrigine 300 mg/d	Radiation necrosis, edema
2	74 years/F	Dizziness, gait disorder	Cerebellar convexity left	No	3.7	166	2	No	6 days	Generalized	Levetiracetam 1 g/d	Postoperative hyponatremia
3	56 years/F	Motoric aphasia, gait disorder	Clivus right	No	4.9	1000	N/A	Infarct of the middle cerebellar peduncle	2 days	Focal	Levetiracetam 2 g/d	Preoperative hydrocephalus

## Discussion

In the current study, we analyzed the incidence and clinical presentation of pre- and postoperative seizures in patients with posterior fossa meningioma. Among 44 patients analyzed, seizures were the presenting symptom in two patients, and three patients had seizures after surgery, resulting in a preoperative and postoperative seizure rate of 4.5% and 6.8%, respectively. To our knowledge, this is the first study to investigate the occurrence of seizures in patients with posterior fossa meningiomas.

In patients with brain tumors, the pathophysiology behind seizures is thought to be a combination of tumor mass effect on the epileptogenic cortex, neurotransmitter disruption, and abnormal acid-base balance in the presence of peritumoral edema [16,17]. In addition, seizures may occur after brain surgery and may be triggered primarily by metabolic disturbances, including high or low sodium levels, hypoglycemia, and dehydration [18].

In recent years, many studies have investigated the incidence and risk factors for seizures in meningioma patients [2,7,9,19]. Englot et al. performed a systematic meta-analysis of 39 studies and reported pre- and postoperative seizure rates of 29.2% and 12.3%, respectively [10]. Independent risk factors for preoperative seizures include male gender [9,10], non-skull base location [7,9,10,19], large tumor size [9], peritumoral edema [2,7,10], and increasing patient age [7]. Similarly, non-skull base location [9], left-sided tumor location [2], and procedural complications [7] were independently associated with the occurrence of postoperative seizures in the cited studies.

In general, posterior fossa tumors are not expected to cause seizures because they do not exert a mass effect on the epileptogenic cortex. For this reason, most studies of meningioma-associated seizures focus on patients with supratentorial meningiomas or completely exclude posterior fossa meningiomas [7,10,12-14]. Few studies have investigated the association between seizures and posterior fossa surgery. Lee et al. reported early postoperative seizures in 1.8% of 726 patients with posterior fossa surgery, including one patient with meningioma. The authors identified metabolic acidosis, hyponatremia, and preoperative hydrocephalus as potential causes of seizures [20]. Suri et al. analyzed 511 patients who underwent surgery for posterior fossa lesions and reported a postoperative seizure rate of 5.9%, which was highest in patients with acoustic schwannoma, medulloblastoma, and astrocytoma. They also described an association between sitting position during surgery, pneumocephalus, and postoperative seizures [11].

In the present study, we analyzed the occurrence of pre- and postoperative seizures in patients with posterior fossa meningiomas only. In this subgroup of meningioma patients, the rates of pre- and postoperative seizures were 4.5% and 6.8%, respectively, which is significantly lower than in studies that predominantly analyzed supratentorial meningiomas. Thus, our results show that posterior fossa meningiomas may also be associated with pre- and postoperative seizures, although the risk of epilepsy is low compared to supratentorial meningiomas.

In two patients with posterior fossa meningiomas, seizures were the presenting symptom of the tumor. Both patients had cerebellar meningiomas and were in excellent medical condition (KPS = 100%). Of note, both patients with preoperative seizures were above 60 years of age and thus older than average in our cohort. The lifetime incidence of seizures in the general population is approximately 5% [21], and it has been shown that the risk of seizures increases with patient age [22]. As the patients had no other cerebellar symptoms, it could be speculated that the diagnosis of meningioma was an incidental finding during further clarification of the seizures. In the second patient, a supratentorial part of the tumor was close to the left temporal lobe, which may also have triggered the seizure. Both patients were seizure-free after surgical removal of the meningioma.

Among 44 patients in our cohort who underwent tumor resection, three patients developed postoperative seizures. The onset of seizures ranged from two days to 17 months after surgery. In total, two patients had seizures during hospitalization, and the third patient had seizures during long-term follow-up. In these patients, different potential causes for postoperative seizures were identified. The first patient received tumor bed irradiation after surgical removal of a WHO III cerebellar meningioma. The patient developed radiation necrosis and infra- and supratentorial edema, both of which may be associated with seizures, as shown in previous studies [2,7,10,23,24]. Metabolic disturbances are a known cause of postoperative seizures independent of tumor location [18]. In this context, the seizures in the second patient may be related to low sodium levels after surgery (115 mmol/l) [25]. The third patient had a large clival meningioma with significant brainstem compression and subsequent hydrocephalus. Similarly, Lee et al. described an association between obstructive hydrocephalus and postoperative seizures in patients with posterior fossa tumors [20].

The use of AEDs after brain tumor surgery is controversial in the literature. Some studies have shown a beneficial effect of prophylactic AEDs in reducing the risk of postoperative seizures, especially in the early postoperative period [19,26,27]. Islim et al. reported that especially patients with frontoparietal and convex tumor locations benefited from postoperative antiepileptic medication, reducing the epilepsy rate by 40% [19]. In other studies, the use of prophylactic antiepileptic medication did not have a significant effect on the postoperative seizure rate [10,28]. At our institution, we do not prescribe prophylactic antiepileptic drugs and use AEDs only for therapeutic purposes. In the current study, all three patients with postoperative seizures received AEDs, achieving complete seizure control in all cases.

Based on our experience and considering the low incidence of postoperative seizures after meningioma surgery, we do not recommend antiepileptic prophylaxis for patients with posterior fossa meningiomas. However, we advocate the use of AEDs in patients with postoperative seizures to achieve seizure control for at least three months.

Limitations

The main limitations of our study are related to its retrospective, single-center design. Therefore, the patient sample is relatively low, and we cannot exclude a potential selection bias. Moreover, since the incidence of pre- and postoperative seizures was low, we did not perform statistical analyses to identify potential risk factors for seizures related to posterior fossa meningioma.

## Conclusions

Among 44 patients with posterior fossa meningiomas, we identified two patients in whom seizures were the presenting symptom of the tumor and three patients who developed postoperative seizures. Possible causes of postoperative seizures in these patients were radiation necrosis and edema, low sodium levels, and hydrocephalus. Seizure control was achieved with AEDs in all patients with postoperative epilepsy. In general, the incidence of seizures in patients with posterior fossa meningiomas is low, so we do not recommend the routine use of antiepileptic prophylaxis.
